# Delayed Presentation of Pruritic Urticarial Papules and Plaques of Pregnancy in the Postpartum Period: A Case Report

**DOI:** 10.7759/cureus.91581

**Published:** 2025-09-04

**Authors:** Aravinda Hariram, Toluwalase Sola-Ogunniyi

**Affiliations:** 1 Obstetrics and Gynecology, Royal Surrey County Hospital, Guildford, GBR

**Keywords:** and plaques of pregnancy, itching in pregnancy, polymorphic eruption of pregnancy, pruritic urticarial papules and plaques of pregnancy, urticaria

## Abstract

Pruritic urticarial papules and plaques of pregnancy (PUPPP), also known as polymorphic eruption of pregnancy, is a benign inflammatory dermatosis that typically presents in primigravid women during the third trimester of pregnancy. Postpartum onset is rare and often underrecognized. We report a case of a 29-year-old primigravida with a BMI of 38 kg/m^2 ^who delivered a healthy 4 kg male infant via emergency cesarean section for fetal distress. On postpartum day five, she developed a pruritic rash on the abdomen, initially suspected to be a drug reaction to low-molecular-weight heparin. Given the short duration of low molecular weight heparin (LMWH) exposure and persistence of symptoms after discontinuation, a drug-induced eruption was deemed unlikely. The rash progressed to the thighs and buttocks. Dermatologic examination revealed erythematous papules and urticarial plaques over abdominal striae with periumbilical sparing, typical of PUPPP. Laboratory evaluation was unremarkable, apart from a mildly elevated C-reactive protein (CRP), which was measured to assess for systemic inflammation or infection, neither of which was clinically evident. A clinical diagnosis of postpartum-onset PUPPP was made. Treatment with oral antihistamines resulted in a rapid resolution within two weeks. The patient was followed for four weeks after resolution, with no recurrence of symptoms. Although rare in the postpartum period, PUPPP should be considered in the differential diagnosis of pruritic eruptions following delivery. Classic distribution patterns, periumbilical sparing, and absence of systemic involvement are key diagnostic features. Risk factors for PUPPP include primigravidity, multiple gestation, excessive maternal weight gain, and high BMI; in this case, obesity was the primary risk factor identified. Conservative treatment is typically effective. This case underscores the importance of recognizing postpartum-onset PUPPP and differentiating it from drug reactions or other dermatoses. Awareness of its clinical presentation allows for timely diagnosis, appropriate reassurance, and effective symptom management.

## Introduction

Pruritic urticarial papules and plaques of pregnancy (PUPPP), also known as polymorphic eruption of pregnancy, is a benign but distressing dermatosis most commonly affecting primigravid women in the third trimester. It presents as erythematous, urticarial papules and plaques that typically begin on the abdomen and may spread to the thighs, buttocks, and trunk [1]. Although the condition is generally self-limiting, patients often report intense pruritus that significantly impacts their quality of life. The exact etiology of PUPPP remains unclear, though theories suggest a combination of hormonal, immunologic, and mechanical factors. It is a common distinct clinical entity with an estimated incidence of one in 130-300 pregnancies [1].

While PUPPP most frequently develops during late pregnancy, its occurrence in the postpartum period is rare but documented. Other postpartum dermatoses that may mimic its presentation include pemphigoid gestationis, atopic eruption of pregnancy, drug-induced exanthems, and infectious eruptions. Diagnosis is primarily clinical, supported by typical distribution patterns, such as periumbilical sparing, which helps distinguish PUPPP from pemphigoid gestationis, where the umbilical area is often involved, and the absence of lesions on the face, groin, and extremities [2]. Conservative management with topical corticosteroids and oral antihistamines is usually effective, and systemic corticosteroids may be considered in severe cases [3].

We present a case of a 29-year-old woman with a low-risk antenatal course, who developed a characteristic PUPPP rash on postnatal day five following an emergency cesarean section for fetal distress. The clinical presentation mimicked a drug-related allergic reaction but exhibited hallmark features of PUPPP, including periumbilical sparing and absence of systemic involvement. Prompt recognition and conservative management led to complete resolution of symptoms within one week. This case highlights the importance of considering PUPPP in the differential diagnosis of postpartum rashes and reinforces that, although uncommon, PUPPP can present de novo after delivery.

## Case presentation

A 29-year-old primigravid European woman with a body mass index (BMI) of 38 kg/m² and no significant past medical history underwent an emergency cesarean section at term for fetal distress, delivering a healthy 4 kg infant. Her antenatal course had been low-risk, with no comorbidities, though she had gained more than 15 kg during pregnancy. She was not on any regular medications prior to or following delivery, except for prophylactic low-molecular-weight heparin (dalteparin) administered postoperatively.

On postpartum day five, the patient noticed the sudden onset of a pruritic rash over her abdomen. Suspecting an allergic reaction to dalteparin, the medication was discontinued. However, the rash continued to worsen and progressively spread to the thighs and buttocks. She denied any history of similar rashes in the past and reported no known drug allergies.

On physical examination, the patient was afebrile and hemodynamically stable. Dermatologic examination revealed erythematous papules and urticarial plaques over the abdominal striae, with characteristic periumbilical sparing. Similar lesions were noted over the thighs and buttocks. There were no vesicles, pustules, or mucosal involvement. The face, palms, soles, groin, and axillae were unaffected.

The baseline blood investigations are shown in Table 1. Blood picture showed mild anemia, which is common postcesarean section from intraoperative blood loss, mild leukocytosis, which is physiological in the postpartum period, and the mildly raised eosinophil count could be due to the skin condition. Lymphocytes and platelet counts are within normal limits. CRP is elevated at 10 mg/L, suggesting active inflammation. Given the clinical features and typical distribution of the rash, a diagnosis of pruritic urticarial papules and plaques of pregnancy (PUPPP) was made, despite the unusual timing of onset in the postpartum period.

**Table 1:** Baseline blood investigations.

Tests	Result	Reference range
Hemoglobin	10.8 g/dL	11.5-15.5 g/dL
White blood cell count	11.5×10⁹/L	4.0-11.0×10⁹/L
Neutrophils	7.5×10⁹/L	2.0-7.0×10⁹/L
Lymphocytes	2.6×10⁹/L	1.0-3.5×10⁹/L
Eosinophils	0.6×10⁹/L	0.0-0.5×10⁹/L
Platelets	320×10⁹/L	150-450×10⁹/L
C-reactive protein (CRP)	10 mg/L	<5 mg/L

**Figure 1:**
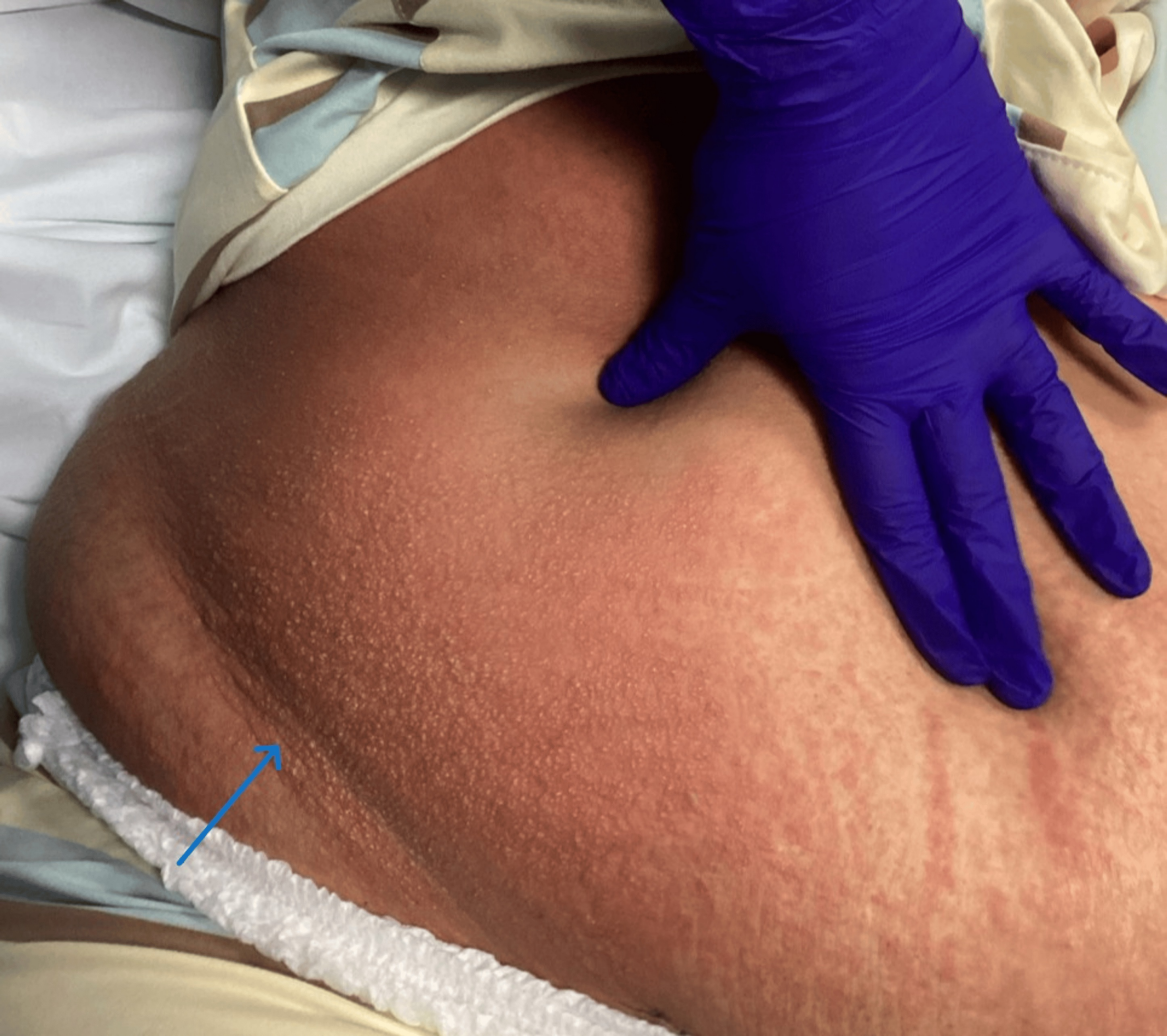
Multiple erythematous urticarial papules coalescing into plaques, predominantly over the striae, causing intense pruritus. The arrow shows erythematous plaques on the lower abdomen.

**Figure 2:**
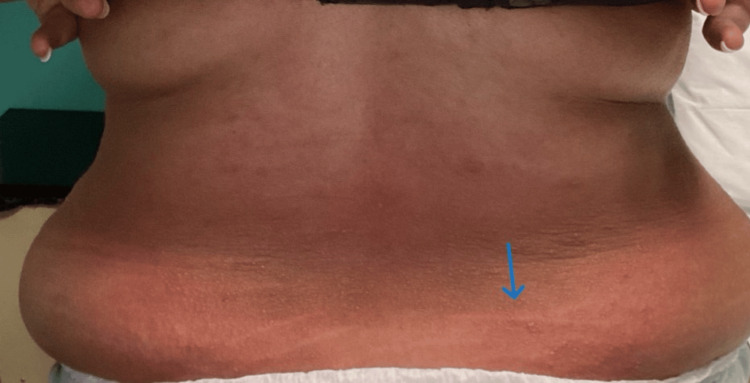
Note lesions extending to the back and excoriations from scratching. The arrow shows excoriation from scratching.

This patient exhibited characteristic features, including pruritic, erythematous papules and urticarial plaques (Figure 1), which extended to the lateral torso and lower back (Figure 2). The patient was counseled about the benign nature of the condition and its typical course of progression. She was started on oral antihistamines for symptomatic relief. At follow-up on day 14 postpartum, the pruritus had completely resolved, with no residual hyperpigmentation.

## Discussion

Pruritic urticarial papules and plaques of pregnancy (PUPPP), or polymorphic eruption of pregnancy, is a benign dermatosis that primarily affects primigravid women in the third trimester. It typically resolves spontaneously postpartum and is rarely observed after delivery. The precise etiology of PUPPP remains unclear, though multiple theories have been proposed. These include abdominal skin distension, hormonal changes, connective tissue damage within striae, immune reactions to fetal DNA (fetal microchimerism), and fibroblast proliferation induced by placental hormones [3]. A strong association with multiple gestations, excessive maternal weight gain, cesarean delivery, and male fetuses has also been documented [4].

In the present case, a 29-year-old primigravida with a BMI of 38 kg/m^2^ and a weight gain of over 15 kg during pregnancy developed a classic PUPPP eruption on postpartum day five, following a cesarean section for fetal distress. The patient delivered a 4 kg male infant; both macrosomia and male fetal sex are known risk factors for PUPPP. While PUPPP typically begins in the late third trimester, this case supports reports suggesting that abrupt hormonal and physical changes associated with labor and cesarean delivery may also play a triggering role [5].

The pathophysiology of postpartum-onset PUPPP is even less well understood than its antenatal counterpart. One hypothesis is that sudden loss of abdominal distension following delivery may expose previously stretched connective tissue to immune activation [6]. Additionally, circulating fetal DNA that persists in maternal skin may provoke an immune response after delivery when immune tolerance mechanisms decline.

The diagnosis of PUPPP is largely clinical. This patient exhibited characteristic features, including pruritic, erythematous papules and urticarial plaques. The papules were mainly present on the striae with periumbilical sparing. The absence of mucosal involvement or palmoplantar lesions further supported this diagnosis [7].

Other dermatoses and conditions considered in the differential diagnosis included the following: drug eruptions(e.g., from dalteparin), which were unlikely given ongoing progression after cessation of the drug. Pemphigoid gestationis, which is usually vesiculobullous and confirmed by direct immunofluorescence (not performed here as the clinical picture was not suggestive). Atopic eruption of pregnancy, which typically begins earlier in gestation and lacks periumbilical sparing. Viral exanthems were excluded based on the absence of systemic symptoms or mucocutaneous involvement. Intrahepatic cholestasis of pregnancy (ICP) was unlikely, as it typically presents with generalized pruritus without rash, and the patient's liver function tests and bile acids were within normal limits. Infectious causes, which were ruled out by normal vital signs, clinical stability, and absence of systemic signs of infection [8].

Laboratory workup revealed a mildly elevated C-reactive protein (CRP) level of 10 mg/dL and mild eosinophilia of 0.6x10^9^/L, likely reflecting localized inflammation. No biopsy was performed, as the clinical diagnosis was clear and non-invasive management was appropriate. The patient was managed conservatively with oral antihistamines, resulting in full symptomatic resolution within two weeks. This aligns with most PUPPP cases, which respond well to topical corticosteroids and antihistamines. In more severe or refractory cases, systemic corticosteroids or alternative therapies, such as autologous whole blood (AWB) injections, have been explored [1,9]. AWB, though not used in the present case, has shown immunomodulatory potential and may represent a future treatment modality, particularly for patients concerned about medication use during breastfeeding. This case reinforces the importance of considering PUPPP even in the postpartum period, especially in the presence of classic features and risk factors, such as primigravidity, rapid abdominal stretching, cesarean delivery, and large-for-gestational-age infants. Prompt recognition and conservative management typically result in complete resolution and reassurance to the patient.

## Conclusions

Pruritic urticarial papules and plaques of pregnancy (PUPPP) is a self-limiting dermatosis usually seen in late pregnancy but may, on rare occasions, present de novo in the postpartum period. This case highlights a classic presentation of postpartum PUPPP in a primigravida with risk factors, including excessive weight gain, cesarean delivery, and a large-for-gestational-age male infant. Early recognition of hallmark clinical features, particularly periumbilical sparing and the absence of mucosal or systemic involvement, allowed for an accurate diagnosis without the need for invasive investigations. Conservative management with oral antihistamines resulted in rapid resolution, and the patient experienced a favorable prognosis with no long-term sequelae. Awareness of postpartum-onset PUPPP is essential to avoid misdiagnosis and unnecessary discontinuation of critical medications, as initially occurred with dalteparin in this case. Differentiation from more serious dermatoses, such as pemphigoid gestationis, or drug-induced eruptions, remains a key diagnostic step. Although recurrence in future pregnancies is uncommon, risk modification through weight control and close dermatologic surveillance may be beneficial. Finally, this case contributes to the limited literature on postpartum-onset PUPPP, underscoring the need for clinician awareness to ensure prompt diagnosis and effective, conservative management.
